# DeepCBS: shedding light on the impact of mutations occurring at CTCF binding sites

**DOI:** 10.3389/fgene.2024.1354208

**Published:** 2024-02-23

**Authors:** Yiheng Wang, Xingli Guo, Zhixin Niu, Xiaotai Huang, Bingbo Wang, Lin Gao

**Affiliations:** School of Computer Science and Technology, Xidian University, Xi’an, China

**Keywords:** CTCF, insulated neighborhoods, liver cancer, proto-oncogene, deep learning

## Abstract

CTCF-mediated chromatin loops create insulated neighborhoods that constrain promoter-enhancer interactions, serving as a unit of gene regulation. Disruption of the CTCF binding sites (CBS) will lead to the destruction of insulated neighborhoods, which in turn can cause dysregulation of the contained genes. In a recent study, it is found that CTCF/cohesin binding sites are a major mutational hotspot in the cancer genome. Mutations can affect CTCF binding, causing the disruption of insulated neighborhoods. And our analysis reveals a significant enrichment of well-known proto-oncogenes in insulated neighborhoods with mutations specifically occurring in anchor regions. It can be assumed that some mutations disrupt CTCF binding, leading to the disruption of insulated neighborhoods and subsequent activation of proto-oncogenes within these insulated neighborhoods. To explore the consequences of such mutations, we develop DeepCBS, a computational tool capable of analyzing mutations at CTCF binding sites, predicting their influence on insulated neighborhoods, and investigating the potential activation of proto-oncogenes. Futhermore, DeepCBS is applied to somatic mutation data of liver cancer. As a result, 87 mutations that disrupt CTCF binding sites are identified, which leads to the identification of 237 disrupted insulated neighborhoods containing a total of 135 genes. Integrative analysis of gene expression differences in liver cancer further highlights three genes: ARHGEF39, UBE2C and DQX1. Among them, ARHGEF39 and UBE2C have been reported in the literature as potential oncogenes involved in the development of liver cancer. The results indicate that DQX1 may be a potential oncogene in liver cancer and may contribute to tumor immune escape. In conclusion, DeepCBS is a promising method to analyze impacts of mutations occurring at CTCF binding sites on the insulator function of CTCF, with potential extensions to shed light on the effects of mutations on other functions of CTCF.

## 1 Introduction

3D genomics is a rapidly growing field that investigates the complex folding and organization of chromosomes in eukaryotic cells. Various techniques have been developed to study the 3D structure of chromosomes, such as 3C (Dekker et al., 2002), Hi-C (Lieberman-Aiden et al., 2009), ChIA-PET (Fullwood et al., 2009), 4C (Simonis et al., 2009), and 5C (Dostie et al., 2006). The three-dimensional structure of chromosomes includes chromosomal domains, chromosome compartments, topologically associated domains, insulator regions, and promoter-enhancer loops. In 3D genomics, insulated neighborhood is defined as a CTCF-CTCF homodimer that binds with cohesions and contains at least one gene’s chromatin loop (Dowen et al., 2014). Miao have observed that this chromatin loop serves as a unit of gene regulation (Yu and Ren, 2017). When the CTCF binding site is disrupted, improper enhancer-promoter interactions can lead to the dysregulation of local genes (Hnisz et al., 2016b).

Recently, a study has revealed that mutations in CTCF binding sites occur frequently in cancer (Katainen et al., 2015). And CTCF/cohesin binding sites are a major mutational hotspot in the cancer genome. Some of these mutations can cause a decrease in CTCF binding, leading to the disappearance of insulated neighborhoods (Hnisz et al., 2016b; Umer et al., 2016). Non-coding mutations at CTCF binding sites have the potential to disrupt insulated neighborhoods, leading to altered gene expression within these regions. This, in turn, could potentially contribute to the development of diseases. Previous research has indicated that there are typically silent proto-oncogenes within insulated neighborhoods. Additionally, the anchoring regions of insulated neighborhoods containing proto-oncogenes undergo frequent somatic mutations in various types of cancer (Hnisz et al., 2016a). In summary, variations in CTCF binding sites in cancer may lead to the disappearance of insulated neighborhoods and the activation of oncogenes, ultimately promoting the development of cancer. So the identification of variants that have the potential to disrupt insulated neighborhood is a critical task.A few studies focous on this task. Zhang et al. has proposed a method, named CTCF-MP, to predict chromatin loops. This method utilizes a machine learning model based on word2vec and boosted trees (Zhang et al., 2018). CTCF-MP algorithm incorporates sequence variations caused by mutations and enables prediction of the influence of such mutations on the formation of chromatin loops. Sequence-based deep learning methods have shown great potential in predicting the impact of genetic variants on insulated neighborhoods. When provided with a pair of DNA sequences of anchors, this model generates a value ranging from 0 to 1, which can be used to determine the probability or strength of the chromatin loop (Zhang et al., 2018). DeepCTCFLoop takes a pair of DNA sequence containing CTCF motifs with flanking regions and encodes it into one-hot encoding as input, uses a neural network to predict whether this pair of sequences can form a DNA loop (Kuang and Wang, 2021). DeepMILO, a deep learning framework, utilizes one-hot encoding to represent DNA sequences, comprises of an anchor model and an anchor orientation model. It accurately predicts the effects of variants on CTCF/cohesion mediated insulator loops and reveals a novel mechanism for oncogene dysregulation in malignant lymphoma (Trieu et al., 2020).

However, CTCF is a multifunctional protein, associated with a number of vital cellular processes such as transcriptional activation, repression, insulation, imprinting and genome organization (Oh et al., 2017). CTCF not only regulates gene expression by forming loops but also can independently regulate gene expression.

While the discussed methods, such as DeepMILO, have certain limitations as they require paired data, making them effective in predicting the impact of mutations occurring at CTCF binding sites on their insulator function. These methods are not capable of predicting the effects of mutations at CTCF binding sites on other function.

Both DeepMILO and DeepCTCFLoop utilize the one-hot encoding method. One-hot encoding treats each position in the sequence as an independent feature, disregarding the sequential relationships between adjacent nucleotides. However, biological sequences often contain important sequence patterns or motifs that play a critical role in the functionality or structure of the sequence. In comparison, using only one-hot encoding may not fully capture the information conveyed by these patterns. In a recent study, a novel method named dna2vec, has been proposed. This method leverages the human genome sequences as the learning corpus and embeds k-mers into a 100-dimensional continuous vector space (Ng, 2017). By employing this encoding approach, the model can capture a more comprehensive set of information, enhancing its ability to capture relevant patterns and features in the sequences.

Considering the limitations of the discussed methods, we have developed a method named DeepCBS, which employs a DNA sequence as input instead of a paired sequence and utilizes the dna2vec encoding method for representation. Applying DeepCBS to somatic mutation data of liver cancer patients, we predicted the impact of these mutations on CTCF binding sites. Then, through analysis of differential gene expression, we identify three potential liver cancer oncogenes, providing potential therapeutic targets for the treatment of liver cancer. In our study, DeepCBS successfully predicts the impact of mutations occurring at CTCF binding sites on insulated neighborhoods. In the future, it can also be utilized to predict the effects of mutations on other functions of CTCF.

## 2 Materials and methods

### 2.1 Data collection and processing

CTCF ChIP-seq data for GM12878, HepG2, K562, MCF-7, and HMEC cell lines is downloaded from the Encode portal (accession: ENCFF710VEH, ENCFF237OKO, ENCFF738TKN, ENCFF738TKN, ENCFF288RFS). We also download RAD21 CHIP-seq raw data of GM12878 (accession: ENCFF002CPK) and CTCF CHIA-PET raw data of GM12878 (accession: ENCFF780PGS). We download comprehensive gene annotation data from GENCODE.

Positive samples are generated by selecting 100 base pairs from the summit of each ChIP-seq peak. Negative samples are generated using the R package gkmSVM by matching the repeat fraction, length, and GC content of the repetitive sequences in positive samples (Ghandi et al., 2016). Then we get 43,631 positive and 48,753 negative samples for GM12878 cell line, 60,229 positive and 56,099 negative samples for HepG2 cell line, 56,889 positive samples and 53,875 negative samples for K562 cell line.

We collect simple somatic mutations data of 1706 liver cancer patients from ICGC database, we also collected RNA-seq data of liver cancer from this database, at the same time (see the Supplementary Material).

### 2.2 Construction of DeepCBS

**FIGURE 1:**
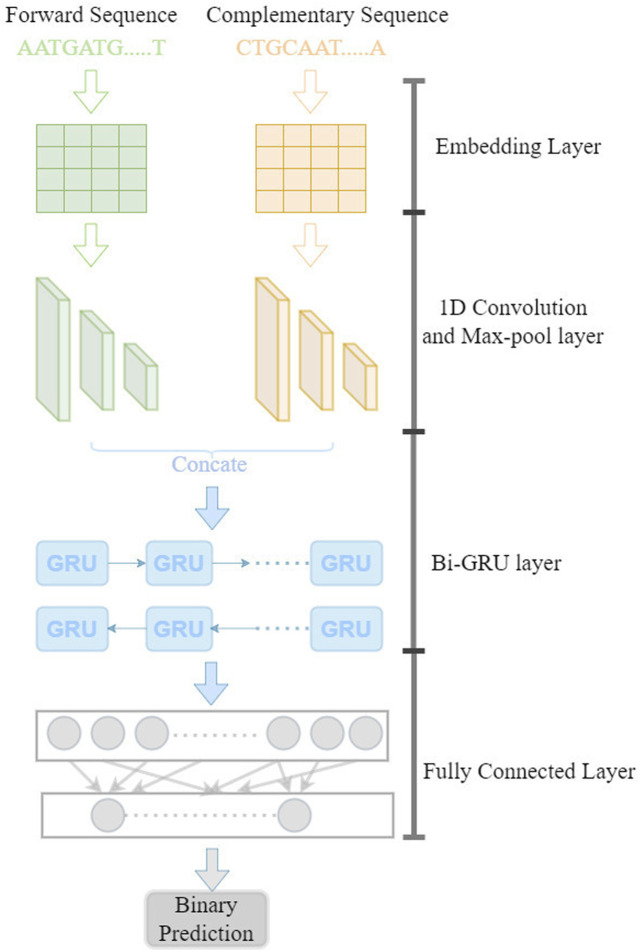
Diagram of DeepCBS.

The model is illustrated in Figure 1. In this model, the forward and reverse DNA sequence with CTCF binding are taken as input by encoding into a matrix using the dna2vec (Ng, 2017) approach. Then, a three-layer convolutional neural network is used to learn the sequence motifs and high level features. The Bi-GRU(Bidirectional Gate Recurrent Unit) layer is used to learn the long-range dependencies between the high-level features. Next, two fully connected layer is used to combine the output from the Bi-GRU layer and make the binary prediction.

### 2.3 Identification of disrupted insulated neighborhoods

We obtain insulated neighborhoods by integrating CTCF ChIA-PET data, CTCF ChIP-seq data, RAD21 CHIP-seq data and comprehensive gene annotation data. Specifically, we define an insulated neighborhood as a CTCF loop whose loop anchors overlap with a CTCF CHIP-seq peak and a RAD21CHIP-seq peak, and which contains at least one gene.

We collect somatic mutation data from liver cancer patients, identify mutated insulated neighborhoods, and utilize the deep learning model proposed in the previous step to predict whether these insulated neighborhoods would be disrupted.

### 2.4 Differential gene expression analysis

We obtain gene expression data from liver cancer patients in ICGC databases. To analyze gene differential expression, we utilized 3 R packages, namely, limma, edgeR, and DESeq2, independently. In order to enhance the robustness of our findings, we obtain differentially expressed genes by taking the intersection of the results from the three packages.

## 3 Results

### 3.1 Workflow of DeepCBS

**FIGURE 2:**
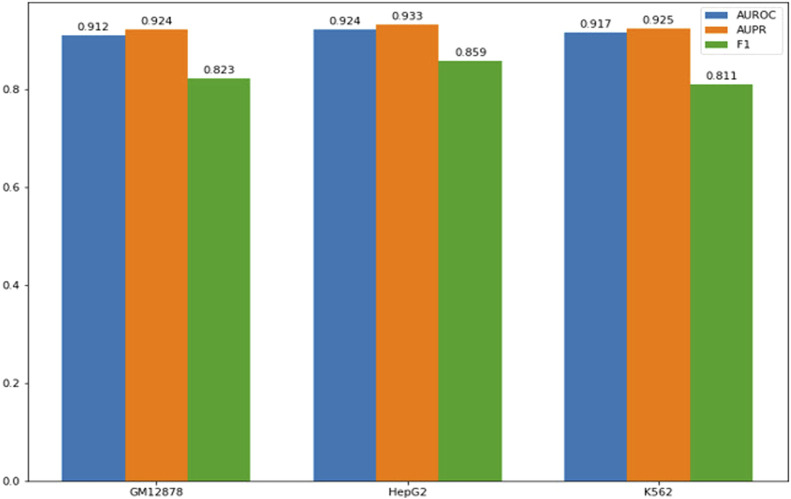
Performance of models on cell type specific CTCF binding sites. The *x*-axis represents the cell lines comprising the training set, while the *y*-axis represents the performance scores.

To elucidate the impact of non-coding mutations occurring at CTCF binding sites, we develop a method named DeepCBS, comprising the following main steps. Initially, we generate positive and negative samples from CTCF ChIP-seq data for 3 cell lines (GM12878, HepG2, K562). Using this data, we train a deep learning model to predict whether mutations on CTCF binding sites lead to the loss of CTCF binding at those sites. Subsequently, we obtain RAD21-mediated loops from RAD21 CHIA-PET data, defining a loop as an insulated neighborhood if both anchors of the loop overlap with CTCF CHIP-seq peaks. And if there are mutations within the CHIP-seq peak region that overlaps with loop anchors, then the insulated neighborhood is considered as a mutated insulated neighborhood. Leveraging the well-trained deep learning model, we predict whether mutations within the mutated insulated neighborhoods disrupt the binding of CTCF, resulting in the disruption of the insulated neighborhooods. In the next step, we observe a significant enrichment of proto-oncogenes in mutated insulated neighborhoods, suggesting that the disruption of these neighborhoods may play a crucial role in cancer development. Consequently, we identify the genes within the disrupted insulated neighborhoods and intersect these genes with the differentially expressed genes in liver cancer. This process yield three genes that may undergo upregulation due to the disruption of insulated neighborhoods. Notably, two out of the three genes have been previously reported as potential oncogenes in liver cancer. The remaining gene, DQX1, is identified as a potential liver cancer oncogene through bioinformatics analysis Figure 2.

### 3.2 Performance of DeepCBS

To assess the model’s performance across different cell types, we employe 1 cell type’s samples as the training data and used samples from other cell types as the testing data, as shown in Table 1. As it can be seen, the model has achieved AUC values over 0.97, demonstrating that our method is a powerful tool for identifying CTCF binding sites.

**TABLE 1:** Cross cell performance evaluation.

Train set	Test set	Auroc
GM12878	HepG2	0.97945
GM12788	K562	0.97364
K562	HepG2	0.97965
K562	GM12878	0.98921
HepG2	K562	0.97507
HepG2	GM12878	0.99106

Since some CTCF binding sites are cell-type specific, we collect CTCF CHIP-seq data from the MCF-7 cell line (breast cancer cell line) and HMEC cell line (normal breast epithelial cell line), and get cell-type specific CTCF binding sites in the MCF-7 cell line. As shown in Figure 2, our models have demonstrated excellent performance on cell-type-specific binding sites.

### 3.3 Identification of potential oncogenes in disrupted insulated neighborhoods in liver cancer

We apply a hypergeometric distribution test to our data and find a significant enrichment of proto-oncogenes in mutated insulated neighborhoods (*p* < 0.05). We have also observed this phenomenon in the data provided by Ji (Ji et al., 2016). This suggests that the disruption of insulated neighborhoods may be a key driver of cancer development, as it can lead to the abnormal activation of proto-oncogene into oncogene. We identify 237 disrupted insulated neighborhoods, comprising a total of 135 genes. We perform differential gene expression analysis, then identify 1,218 differentially expressed genes using 3 R packages. To explore which genes among the 135 affected genes in the disrupted insulated neighborhoods are key genes related to cancer. Then, we take the intersection of the differentially expressed genes with the genes located within the disrupted insulated neighborhoods, which resulted in the identification of three key genes: ARHGEF39, UBE2C, and DQX1. And all of them are upregulated genes, potentially activated due to the disruption of insulated neighborhoods.

ARHGEF39 is a novel member of the Dbl-family of guanine nucleotide exchange factors (Wang et al., 2012). Guanine nucleotide exchange factors are recognized as crucial activators of Rho GTPases, which play a significant role in cell migration (Cook et al., 2014; Goicoechea et al., 2014). Overexpress of ARHGEF39 promotes gastric cancer cell proliferation and migration through the Akt signaling pathway (Wang et al., 2018; Zhou et al., 2018). Previous literature has proposed that ARHGEF39 may act as an oncogene in the progression of liver cancer, and thus represents a potential prognostic indicator and therapeutic target for this disease (Gao and Jia, 2019). Ubiquitin-conjugating enzyme E2C(UBE2C), a member of the E2family, is encoded by the UbcH10gene situated on human chromosome20q13.12. Its function involves the degradation of various target proteins through catalysis. UBE2C has been found to be upregulated in various types of cancer, including breast cancer, and is considered a potent proto-oncogene associated with tumor malignancy (Chou et al., 2014; Han et al., 2015). In liver cancer, UBE2C has been identified as a potential oncogene that can promote cell proliferation, migration, invasion, and drug resistance (Xiong et al., 2019).

Based on the above, we speculate that in liver cancer, the overexpression of ARHGEF39 and UBE2C serves as activated oncogenes and is involved in liver cancer development due to the disruption of the insulated neighborhoods containing them. However, there is currently no literature exploring the role of the DQX1 in liver cancer.

### 3.4 Overexpression of DQX1 is oncogenic in liver cancer

**FIGURE 3:**
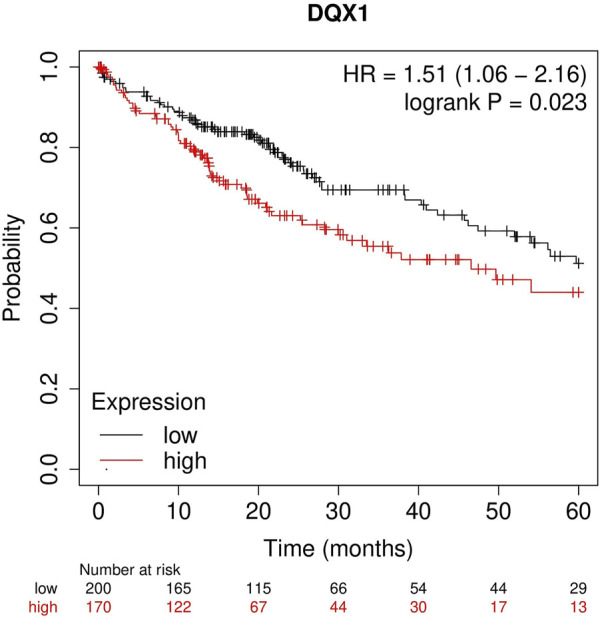
Correlation between DQX1 gene expression and survival prognosis of liver cancer.

The Kaplan-Meier plotter (https://kmplot.com/analysis/) is a powerful tool that enables the assessment of the impact of 54k genes (including mRNA, miRNA, and protein) on survival across 21 types of cancer (Győrffy, 2023). In this study, we focus on the analysis of the relationship between DQX1 expression and survival in liver cancer. The result of survival analysis, as shown in Figure 3, show that highly expressed DQX1 is linked to poor prognosis of overall survival (OS) for cancers of liver cancer.

**FIGURE 4:**
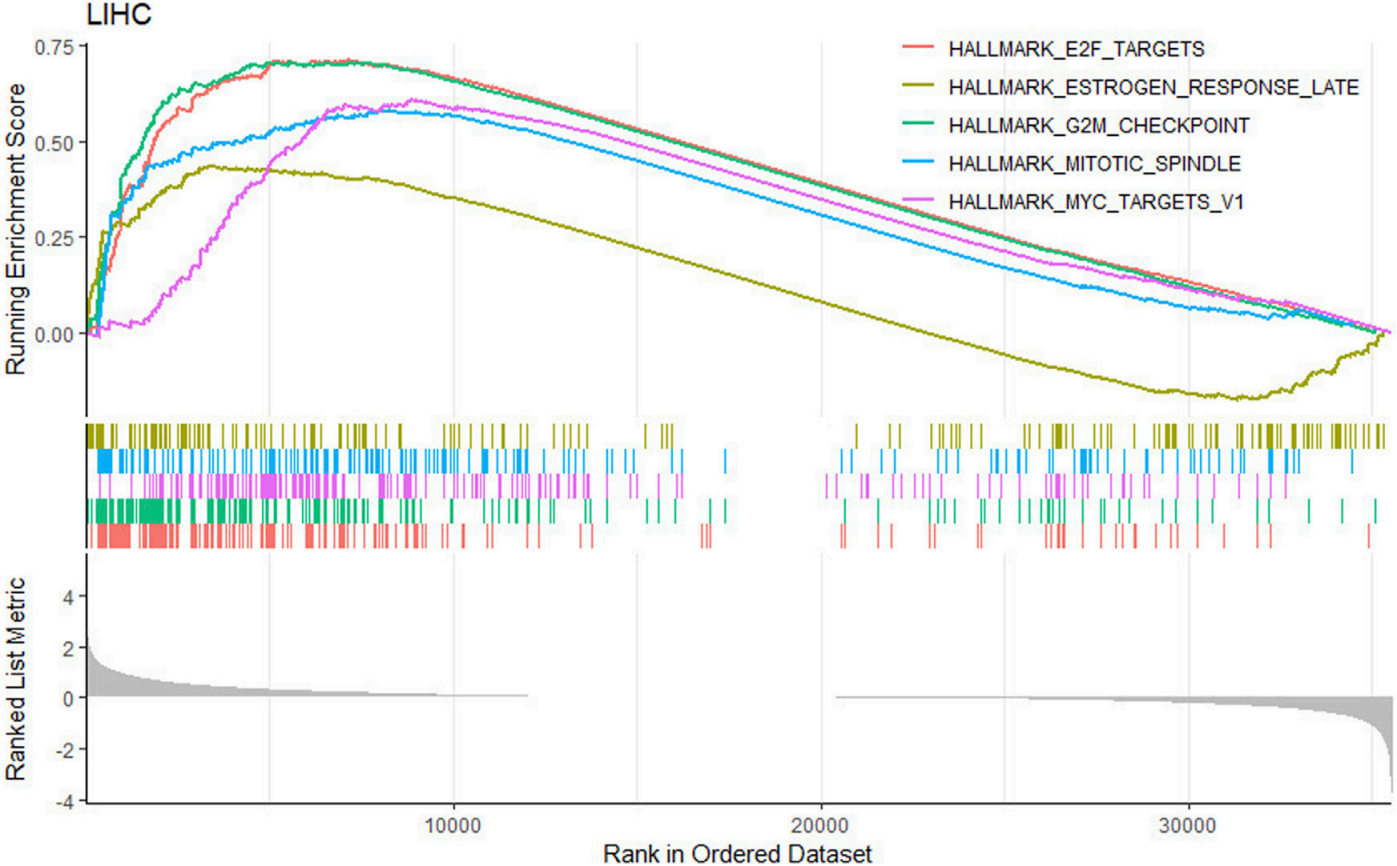
Signaling pathways associate with DQX1 in liver cancer. The depicted signaling pathways in the figure are all cell proliferation-related pathways.

Gene set enrichment analysis (GSEA) is further performed to explore the signaling pathways and molecular mechanisms that were differentially affected by DQX1 in liver cancer. In this study, the tumor samples are grouped based on the mean expression level of DQX1. Samples with expression levels higher than the mean are assigned to the high-expression group (DQX1. Hi), while those with expression levels lower than the mean are assigned to the low-expression group (DQX1. Low). In our study, the Hallmark database is utilized for performing the gene set enrichment analysis. As depicted in Figure 4, the analysis reveal that high expression of DQX1 is significantly associated with the activation of cell proliferation-related pathways. This finding suggests that DQX1may play a crucial role in promoting cell proliferation in liver cancer.

DQX1 is one of the RNA-binding protein genes and RNA-binding protein can regulate the infiltration degrees of immune cells (Sun et al., 2021). Therefore, we implement an immunological

**FIGURE 5:**
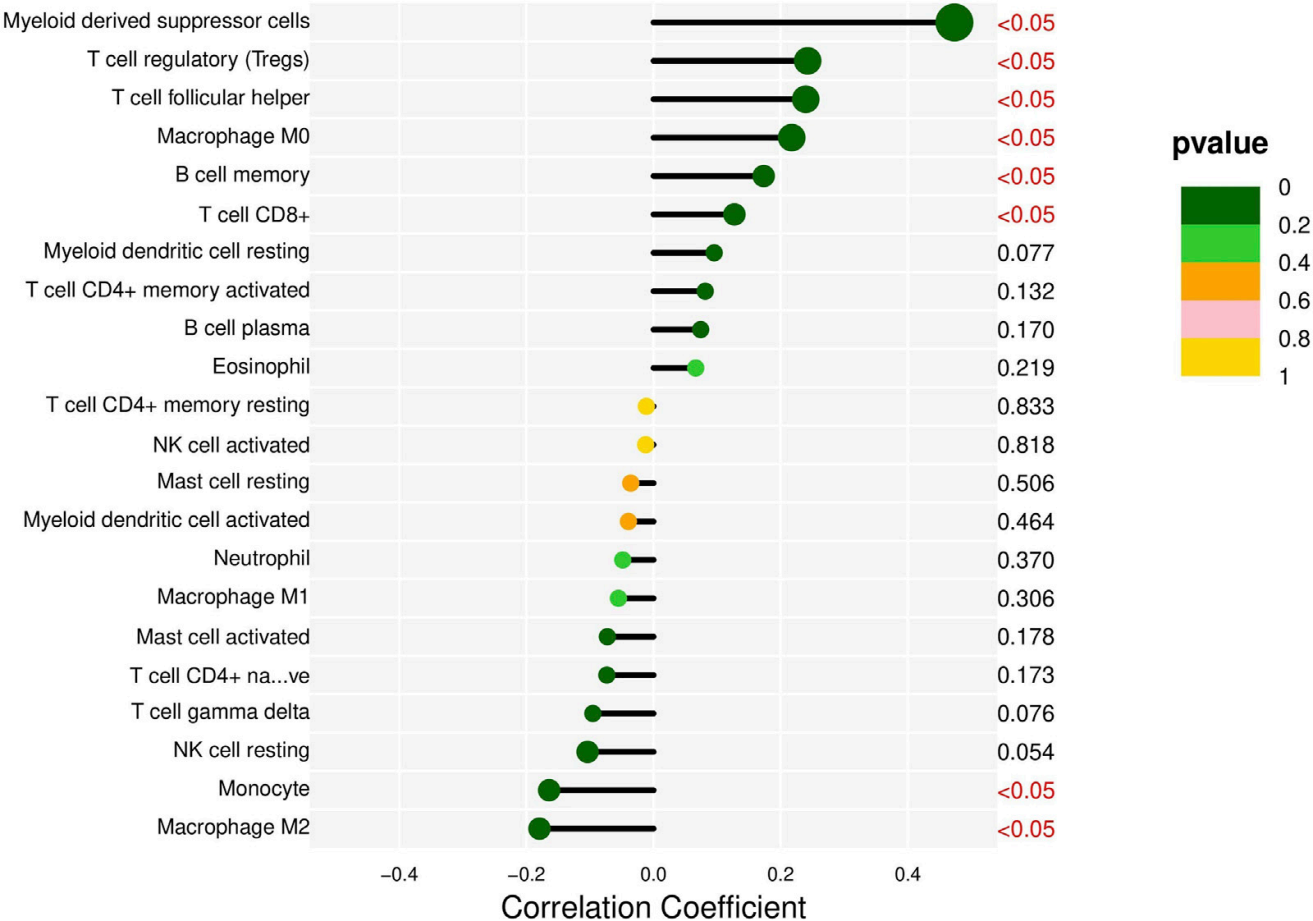
The correlation between DQX1 and immune cell infiltration in liver cancer.

Analysis of DQX1 in liver cancer. TIMER, a comprehensive online resource for the systematic analysis of immune infiltrates in various cancer types, is employed in this study to explore the correlation between DQX1 expression in liver cancer and different immune infiltrates (Li et al., 2020). The results are presented in Figure 5.

**FIGURE 6:**
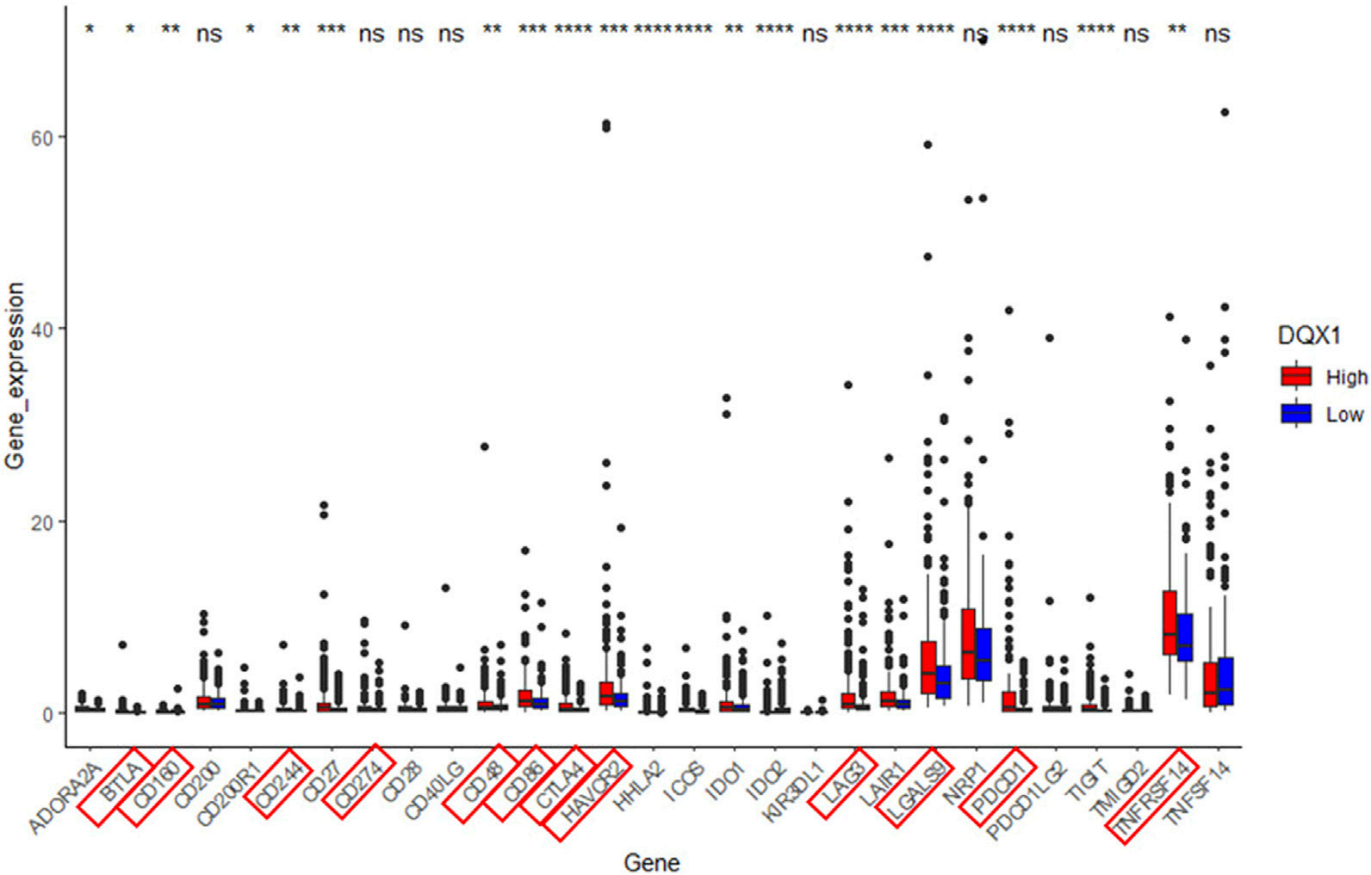
Significant analysis of differential expression of immune checkpoint genes between the high-expression group (DQX1. High) and the low-expression group (DQX1. Low). The markers enclosed within the red box represent co-suppressive immune checkpoints. ***p < 0.001, **p < 0.01, *p < 0.05. ns: non-significant differences (*p* > 0.05).

Also, we perform differential expression analysis of immune checkpoint genes in relation to DQX1 using the same grouping approach as in GSEA. The result is depicted in Figure 6.

Overall, the expression of DQX1 shows significant positive correlations with immune infiltration levels of regulatory T cells (Tregs), myeloid-derived suppressor cells (MDSCs), and expressions of co-suppressive immune checkpoints, contributing to immune escape. This suggests that we can develop immunotherapies targeting DQX1 for the treatment of liver cancer, in the future.

Based on the bioinformatics analysis, we have been inferred that DQX1 may potentially act as an oncogene and be involved in the development of liver cancer.

## 4 Conclusion

In summary, the CTCF play an crucialrole in maintaining these insulated neighborhoods. The disruption of CTCF binding sites can lead to dysregulation of contained genes, potentially resulting in the activation of oncogenes and promoting cancer development. It is important to shed light on the impact of mutations occurring at CTCF binding sites. So we develop a novel method, DeepCBS, to analyze the impact of mutations occurring at CTCF binding sites. Our analysis has identified three potential oncogenes, ARHGEF39, UBE2C, and DQX1 of liver cancer. All three genes play an oncogenic role in the development of liver cancer. And overexpression of DQX1 is associated with poor prognosis and tumor immune escape. Our findings demonstrate the potential of DeepCBS to analyze the impact of mutations occurring at CTCF binding sites, as well as providing valuable insights for the diagnosis and treatment of liver cancer. Over all, this study emphasizes the importance of understanding the 3D organization of the human genome and its impact on gene regulation, as well as highlights the potential of computational methods to identify new targets for cancer therapy.

## Data Availability

The original contributions presented in the study are included in the article/Supplementary Material, further inquiries can be directed to the corresponding author.
