# Supplementary material for: DeepCBS: shedding light on the impact of mutations occurring at CTCF binding sites
Source: Front Genet. 2024 Feb 23;15:1354208. doi: 10.3389/fgene.2024.1354208 (PMC10920299; doi:10.3389/fgene.2024.1354208)
Supplement: Supplementary file 1 [file Table1.DOCX]

Supplementary Material

# DeepCBS: Shedding Light on the Impact of Mutations Occurring at CTCF Binding Sites

Yiheng Wang ^1^, Xingli Guo ^1*^, Zhixin Niu ^1^, Xiaotai Huang ^1^, Bingbo Wang ^1^, Lin Gao^1^

^1^School of Computer Science and Technology, Xidian University, Xi’an, China.

***** Xingli Guo
xlguo@mail.xidian.edu.cn

# Supplementary Data

The somatic mutation data and RNA-seq data for liver cancer patients is available for download through the following link:

[Liver Cancer - CN](https://dcc.icgc.org/projects/LICA-CN) (402 donors)

[Liver Cancer - FR](https://dcc.icgc.org/projects/LICA-FR) (252 donors)

[Liver Cancer – Hepatocellular macronodules](https://dcc.icgc.org/projects/LIHM-FRhttps:/dcc.icgc.org/projects/LIHM-FR) (4 donors)

[Liver Cancer – NCC,JP](https://dcc.icgc.org/projects/LINC-JP) (394 donors)

[Liver Cancer – RIKEN,JP](https://dcc.icgc.org/projects/LIRI-JP) (258 donors)

[Benign Liver Tumor FR](https://dcc.icgc.org/projects/LIAD-FR) (32 donors)

[Liver Hepatocellular carcinoma – TCGA,US](https://dcc.icgc.org/projects/LIHC-US) (364 donors)
